# Are physicians’ strikes ever morally justifiable? A call for a return to tradition

**DOI:** 10.4314/pamj.v6i1.69081

**Published:** 2010-08-21

**Authors:** Mawere Munyaradzi

**Affiliations:** 1 Universidade Pedagogica,Faculty of Social Sciences, Department of Humanities, CP49, Xai-xai, Mozambique

**Keywords:** Physicians,, Physicians,, strike,, medical fraternity,, African traditions

## Abstract

Though physicians strike provides an opportunity to generate more knowledge about the process in which legitimacy of an organization can be restored, it meets with a great deal of resistance not only by the public but from within the medical profession. This paper critically examines the legitimacy of strike by medical doctors heretofore referred to as physicians. Though critically reflecting on strikes of physicians in general, the paper makes more emphasis on Africa where physician strikes are rampant. More importantly, the paper argues that strike implies a failure for everyone in the organization (including the strikers themselves), not only the responsible government or authority. This is because when a strike occurs, an organization/fraternity is subjected to questions, scrutiny and slander. It becomes difficult to decouple what is said, decided and done. Traditionally, all medical fraternities the world-over are committed to acting comfortably to external demands-guaranteeing the patients’ lives and public health. By paying attention to external reactions, the medical fraternity adapts and learns what ought and should be done so that it is never again caught in the same messy. At the same time, the fraternity prepares itself for the future strikes. When the fraternity and those outside consider it is doing up to the external expectations, its lost legitimacy is restored. When legitimacy is restored, external pressure like once disturbed water returns to normal.

## Introduction

While there is monumental literature on pro-strike arguments on the one hand and con-strike arguments on the other, there is patchy literature that examines the nexus between the preservation of medical professionalism through African traditional beliefs/practices and the safeguard of human rights of the ordinary patients against physician strike actions. At best, academics have conceived these constituencies of academic research as irreconcilable research spheres that are beyond compromise.

Drawing on the conception of physician strikes in Africa and the world-over, this work contributes to this grey area by demonstrating that the pro-strike arguments have been extreme, narrowly focused and riddled with injustices. On the other hand, the anti-strike arguments based on the Hippocratic Oath need reconstruction or reconstitution or both; otherwise they cannot be accepted as well on philosophical grounds.

It is worth noting at this juncture that all physician strike actions take place within an institution/medical fraternity which has a structure, a set of policies, a culture/tradition and a set of factual decision-making. Yet, the fraternity’s tradition and set of policies among other things are violated when a strike occurs. When this happens some blame the physicians and others praise them; hence making strike actions a contested terrain. It is this light that the issue deserves incisive intellectual investigation, especially in an African context where incidences of strikes are rampant.

The intractable nature of physician strike action heretofore referred to as strike makes it at root ethical and, in part, economic. It is in part economic because it deals with a group of professionals who offer their services for a price. And it is at root ethical in so far as it concerns itself with human behavior and conduct and the difference between right and wrong, good and bad. This denotes that strike does not deal solely with factual judgments which can be said to be either true or false; hence its complexity. Consequently, the question on strike has become a common game for almost everybody-moralists, economists, academicians, national governments and the public. It has also become one of the most contentious of contemporary issues in the medical fraternity.

This paper conceptually analyses strike before advancing a con-strike argument based on four views/theories namely: utilitarianism, modified Hippocratic Oath and African communalism and the principle of beneficence. The sacrosanct of human life as is negatively undermined and threatened by physicians strike necessitated the research of this nature. Using the aforesaid theories, the paper makes an attempt to demonstrate through ‘cases’drawn mainly from Africa the plausibility of con-strike view. This view is useful in that it pays veneration to the sanctity of life and promotes the majority’s happiness. It also represents a human right oriented response from a patient and the public’s perspective. More importantly, the emancipator approach of the paper uses ‘exemplary cases’ to demonstrate how we can seek to understand the impact of strikes from credence values of beneficence, mercy and simple logic. This paper therefore is an attempt to integrate African communalism, utilitarianism and the principle of civil rights and a modified version of the Hippocratic Oath into the main stream of physicians’ strike discourse. In most if not all countries, this is necessary because in the name of physicians’ strike, unnecessary deaths and sufferings of the public are inevitable. And civil rights are often violated and neglected, yet there are long term advantages to be gained by actively promoting them.

In short, the virtue of this paper is to ascertain how useful and influential the anti-strike view based on African communalism, the principle of beneficence, utilitarianism and modified Hippocratic Oath is, especially as a strategy where forces of medical ethics would essentially benefit healthy professionals, the public and their national governments in decision making relevant to the medical fraternity. The paper is therefore a contribution towards efforts by those who are against physicians strike. It shifts emphasis from the dominant Western view on physician strike based on the old version of the Hippocratic Oath to the view against physicians strike as understood from the standpoints of African communalism, the principle of beneficence, utilitarianism and a modified Hippocratic Oath.

It should be quickly noted, however, that the answer to the moral problems surrounding physician strike is very difficult to stipulate; thus the role of judging and deciding problems facing physicians should not be solely accredited to physicians alone, nor should it be accredited to the government alone. Instead, many parties such as non-governmental organizations, moralists, members of the public, patient committee, physicians and academics and the national government should contribute before a final deliberation to go on strike is made.

The intricacies surrounding the question of physicians strike can best be disentangled and cogently dealt with only after attention is paid to the concept ‘strike’ itself. This concept thus shall be discussed briefly before looking at the question on the morality and legitimacy of physicians strike; otherwise, the entire task of this paper would tantamount to chasing the wind.

## Conceptual analysis of strike

Strike is an issue in the medical fraternity that has aroused the interest of many professional ethicists, academicians and the public in general. Though oftenly used as a bargaining tactic for most of the workers in the world-over, the concept is deeply controversial, for moral and practical reasons especially when dealt with in relation with physicians or workers in the medical fraternity in general. A number of interpretations to the term have been provided by scholars. Some have generally considered striking as a bargaining tactic by workers. Others have understood it as ‘self contradictory action by’. However, besides these generalizations, various attempts to formulate a workable definition of this obscure term have been made. Marxist-Leninist regimes such as the former USSR or the People’s Republic of China define strike as a counter-revolutionary action-an action by workers against themselves [1]. Contrary to this sense, http://www.direct.gov.uk/en/Employment/TradeUnion/index.htm, defines strike action as a work stoppage caused by the mass refusal of employees to work. According to the same source, strike can also be defined as “industrial dispute” [1,2]. The differences between the definitions above suggest the reason why there is no consensus on the morality and legitimacy of strikes. It can however be argued that these definitions are ambiguous, because they do not make any reference to reasons that give rise to a strike. Also, the definitions does not make reference to who the strike is executed against, hence the need for a more encompassing and comprehensive definition. In this view, I shall define strike as mass refusal of employees to work according to their employment contract in response to an injustice(s) against them by their employer or in response to their grievances with the employer. It is curious to note that strike is one of the two forms (besides action short of strike such as ‘go-slows’, call-outs’ or overtime bans) of industrial action; hence the need to define the latter. Industrial action is an action whereby members of a trade union are involved in a dispute with their employer that cannot be resolved by negotiation [3].

### Clashing views on physician strike

The question on whether physicians should go on strike or not has so far received different interpretations. It is indeed a contested terrain. Different interpretations have been conjured, yet no consensus has been reached. Two main camps have however been prominent since the late 19^th^ and early 20^th^ centuries when most western countries partially legalized striking [4]. While some people are against the idea of physicians going on strike, others support the idea as long as physicians feel they are being unjustly treated by their employer. In the face of such varied modes, it is perhaps unwise to attempt to reflect on the question on physicians’ strike before paying some attention to the views given so far against and in favor of physicians’ strike from whence the aforesaid camps ensue. Yet, it is a truism that only one and not both views for and against physicians’ strike can be right. In is therefore the contention of this work that some of the previous researches on the issue under consideration were erroneous and others inadequate; hence the need to re-examine their assumptions and contributions. In the ensuing paragraphs, two camps on physicians’ strikes, pro-strike and con-strike arguments, thus, shall be discussed briefly and separately in an attempt to determine the most plausible and philosophically convincing position.

### The Pro- Physician Strike View(s)

Negative scholarship on the idea of physicians going on strike has recently emerged. Robert Nozick and Beauchamp, for example, have initiated what unfortunately will be a growing trend of promoting the idea that physicians should go on strike whenever they deem necessary. What is distinctive of these scholars is that their approach is enormously economic. They seem to have been driven by a somewhat biased capitalistic philosophy which undermines morality, religion and all perspectives linked to Afro-centric philosophy. Such philosophers cannot be expected to be genuinely seeking the truth about the ethic-economical issue of physicians’ strike. Beauchamp, for example, argues that physicians should go on strike whenever they deem necessary. In his words:

*“Any system that favors the idea that physicians have ethical obligations that transcend self-interest, exigency and even political and economic forces is evaluated as capricious and unjust in so far as it fails to determine how social burdens and benefit sought to be allocated”* [5].

It is interesting to note that, for Beauchamp, if the government or any system responsible for physicians’ salaries fails to cohere with conventional standards and, to recognize their valuable social contribution, need and ambition, the latter are morally justified to go on strike. For him, strike is a way of reacting against an injustice of social, political, religious or economic nature. To this effect, his argument appeals to the principle of justice; hence the need to understand justice. For purposes of this study, the researcher shall identify with Pellegrino and Thomasma [6] who define justice as the habit of rendering what is due to others. For example, if a patient in a hospital deserves treatment to be cured of his/her disease, justice is done if and only if the patient receives the right treatment at the right time.

The example given above shows that one who has a claim based on justice has a claim of entitlement. In this strong sense, the patient given in the example is due to something. An injustice, in turn, is done if s/he is denied to that s/he is entitled, in this case, treatment. It is in this light that physicians all over the world feel morally justified going on strike whenever they feel they are unjustly being treated by the government or authority responsible for their grievances. Although physicians are few of the most highly paid professionals in Africa and the world-over, those in developing countries still feel shortchanged and unjustly paid; hence involved in strikes most of the time. Yet, questions that worry the critics and even the friends of strike partisans can be posed: What is so special about physicians as professionals that besides being the most highly paid professionals in most countries they still think they are unjustly paid? And is it morally justifiable to pay them ‘hefty salaries’ when the majority of the people (as in the case of most developing countries) are suffering from abject poverty?

To answer the question, we must know what justice is and, by what method/criteria we should judge an action as just. In view of the above raised question, John Rawls who understands justice as “fairness” [7] argues that:

“*What persons are entitled to or can legitimately claim is based on certain morally relevant properties they posses, such as being productive or being in great need (scarce). It is wrong, as a matter of justice, to burden someone if the person possesses the relevant property”* [8].

Transposing this argument to the issue of physicians, it can be argued that physicians posses morally relevant properties insofar as they are a scarce resource, productive and greatly needed. It is out of this understanding that Beauchamp and Rawls argue that physicians are morally justified to go on strike if they are not happy with their wages or conditions of service. A lingering question(s) arises at this juncture: Does the fact physicians have morally relevant properties makes their strike a morally justifiable act? And does the morally relevant properties of physicians justify them gaining economic advantage over other professionals abiding by similar existing societal principles and values?’

Nozick reasons along with Beauchamp and Rawls in support of physician’s strike. He employs a libertarian theory of justice. In his words a government’s action is justified if and only if it protects the rights the rights or entitlements of citizens, the right of citizens not to be coerced [9]. But in the medical fraternity whose rights to respect? Should it be those of physicians or those of patients or both? In view of this question, Nozick is tempted to reason that the rights of both should be preserved; however, those of doctors should be preserved first if justice is to prevail. For him, satisfying the needs and interests of physicians would in turn satisfy those of patients. His argument can be understood through the following  logic: 1) If the needs and interests of physicians are satisfied, physicians will in turn satisfy the interests and needs of the patients; 2) Physicians’ needs and interests are satisfied. Therefore the patients’ needs and interests are satisfied.

It is curious however, to note that Nozick’s argument is a contemporary malaise -one based on haste generalization for it is possible that physicians’ needs and interests are satisfied yet the latter still fails to satisfy the patients’ needs and interests. It is therefore surprising and disturbing that Nozick strongly believes that if government satisfies the needs and interests of physicians, it is acting justly to all citizens, even the patients and the public in general. In this light, the paper contends that physicians strike is never morally good and justifiable on the basis of Nozick’s argument. A critical question can even be raised against Nozick: Is it not that by going on strike, physicians are guilty of the same offence they level against the government or whatever organ responsible for their salaries and working conditions; that of being unjust to their patients and the public?

As has been seen, arguments by strike partisans are unsustainable and philosophically implausible. They are all narrow in that they look at strikes solely from an economic perspective. Yet, the question of strike is at root and indeed largely ethical, and not wholly an economic issue.

Having put to rest arguments by pro-strike partisans as futile in solving the question on morality and legitimacy of physician strike, let us now turn to con-strike arguments – arguments which support the position of this paper. It is the conviction of the paper that these arguments though need either refinement or reconstitution or both offer a solid answer to the question of the morality and legitimacy of physicians strike. The ensuing section shall therefore examine arguments against physicians; modify or reconstruct them where necessary in a bid to demonstrate how formidable arguments against physicians’ strike are. Four arguments; argument based on a modified version of Hippocratic Oath, argument from the principle of beneficence, argument from utilitarianism and argument from African communalism shall be forwarded to reinforce the con-physician strike position.

### The Con-Physician Strike View

Most of the con-strike partisans have forwarded arguments against physicians’ strike based on the ‘old version of the Hippocratic Oath’. For various reasons, as shall be seen, these arguments have been perceived as philosophically implausible and so have faced serious criticisms. For this reason, I shall defend the anti-physician strike view not only using a modified version of the Hippocratic Oath; but three arguments namely; argument from the principle of beneficence, argument from utilitarianism, and argument from African communalism.

### Argument from Hippocratic Oath

Roth [10] defines the Hippocratic Oath as an ethical perspective in ancient Greece which contends that the knowledge of medicine and that of medical ethics is available only to the Hippocratic physicians. The Hippocratic physicians were a group of committed physicians (women and youth excluded) in ancient Greece who would use treatment to help the sick according to their ability and judgment but never with a view to injury and wrongdoing and, sometimes give their services for free [11]. The Oath stresses that only men, and not women can assume the duties of physicians. A number of questions can, however, be raised: Isn’t it that women are naturally more loving and caring than men? And, don’t we have some cases in the medical fraternity that require only women physicians, and not men to handle? It is in the light of these questions that the Hippocratic Oath and all arguments based on it have suffered criticism. And though a formidable challenge to the pro-physician strike partisans, all arguments based on it have been criticized for being sexist, selective, elitistic and paternalistic.

Having made these observations, the researcher of this work argue for the modification of the Hippocratic Oath to include both sexes and age groups (men and women, young and old) before advancing the argument that the Hippocratic Oath (the new version) can act as the source of power and reason for those who argue against physicians’ strike. The Hippocratic Oath, thus, should be understood as an ethical perspective which contends that the knowledge of medicine and that of medical ethics is available to all physicians, [12] men and women, young and old to...help the sick (sometimes free of charge) according to their (physicians) ability and judgment but never with a view to injury and wrongdoing [13].

understanding and a high degree of collective responsibility on all physicians. It clearly acknowledges that physicians must reconcile two opposing orders-one based on the primacy of the covenant with patients and the other based on the ethos of self-interest. In this vein, physicians face the tension between self-interest and altruism, of which, to uphold self-interest by going on strike would turn them into entrepreneurs, businesspersons or agents of fiscal, social or economic policy. It is on the basis of this understanding that it can be argued that physicians (men and women, young and old) are morally obliged not to go on strike, but to display their special knowledge, skills and commitment to principles requiring the use of those skills altruistically. And this social mandate should be grounded at medical school. Otherwise, medicine will be expressed in a fatally distorted way; hence losing its originality and meaning.

It can still be argued on the basis of the Hippocratic Oath that physicians in most if not all countries are trained for free using tax payers’ money and government institution like national hospitals. Their knowledge, therefore, is not proprietary but acquired through the privilege of a medical education. This connotes that by accepting the privilege of a medical education, those who enter into medicine become parties to the Hippocratic Covenant with the society-one that cannot be dissolved unilaterally [14]. In other words, medical students, from their first day, enter into a community bound by a moral covenant; a covenant that calls for full responsibility of stewardship of medical knowledge they obtain at free price. Moreover, this covenant is acknowledged publicly when the physicians take an Oath at graduation [15]. The Oath is a public promise that the new physician understands the gravity of his or her calling and therefore promises to use competence in the interests of the sick and the public-fidelity to patients and the public. In this light, to argue that physicians should go on strike whenever they deem necessary is a violation of the covenant of trust and canons of medical ethics-a gross misunderstanding of the parameters and ethical principles that guide medicine in its proper functioning. In fact, strikes shrink physicians’ professional latitude and diminish their professionalism as such.

### Argument from the Principle of Beneficence

More commonly in biomedicine, beneficence is understood as a principle of medical ethics that requires that physicians benefit as well as prevent and remove harmful conditions from patients [16]. The principle has its source from the Hippocratic Oath. In the light of the definition of beneficence explicated above, I identify with Veatch [17] who argues that:

“*Medicine is at heart a moral community and will always be; that those who practice it are ‘de facto’ members of a moral community bound together by knowledge and ethical precepts; and that, as a result, physicians have a collective as well as individual moral obligation to protect the welfare of sick persons”*.

This is to say that physicians as a group of morally committed persons should always seek the greater balance of good over harm in the care of patients. An important question arises at this juncture: Does physicians’ strike seeks the greater balance of good over harm in the care of patients? It is the conviction of this paper that this can never be. Actually, physicians strike does more harm than good, not only to the patients, but members of the public-it (strike) is in itself a crime against humanity committed by the physicians veneered by relentless pursuit of self-interest motives. This is not to say that physicians are not human beings with interests and needs alike other human beings. However they should understand that their profession is a calling-a serious de facto obligation which requires them to always seek the greater balance of good over harm in the care of patients. In subtle ways, the virtues of a good person and of the good physician overlap [18].

More importantly, it remains a truism that medicine as a profession and members of the public implicitly expect physicians to remain members of a moral community dedicated to altruism other than self-interest. To place self-interest ahead of the interests and needs of the patients would therefore mean converting the medical profession into a trade -a total abnegation of the very essence of what it means to be a physician. It is toleration of practical incompetence and selfish motives with incalculable long-term effects; hence is morally unjustifiable.

### Argument from Utilitarianism

Utilitarianism is a doctrine which states that the rightness or wrongness of an action is determined by the goodness or badness of its consequences [19]. This means that utilitarianism is a consequentialist theory in so far as it calls for the assessment of actions in terms of their ends and consequences, their contribution to happiness and prevention of suffering. In fact, according to utilitarianism, an action is good or right when it achieves the greatest happiness for the greatest number, otherwise it is bad. Kantian ethics, based on the concept of duty, holds that an action is good if it is based on good intention. For utilitarians, an action in itself has no moral worth and takes moral value only when it is considered in conjunction with its effects. To the contrary, Kantians argue that what makes an action right is not its consequence(s) but the fact that it conforms to the moral law [20]. Thus unlike deontological theories which look at the action itself, utilitarianism assess the rightness or wrongness of an individual or group’s action directly by its consequences and nothing else. De George offers some clarification of consequentialist and deontological ethics:

“*One approach argues on the basis of consequences (consequentialist); it states that whether an action is right or wrong depends on the consequences of that action. The second basic approach is called the deontological approach. It states that duty is the basic moral category, and that duty is independent of consequences. An action is right if it has certain characteristics or is of a certain kind, and wrong if it has other characteristics or is of a certain kind”* [21].

Utilitarianism and Kantian ethics are examples of consequentialist and deontological ethics, respectively. For Kant (the representative of Kantian ethics) the moral law or the highest principle of morality is based on human reason.

This work does not seek to undertake a comprehensive discussion of consequentialist and deontological ethics, but to demonstrate the usefulness and plausibility of utilitarianism in criticizing physicians strike. However, any ethical theory that begins from some external demands and consequences faces the challenge of legitimacy. The challenge is that what ought to be done remains foreign to who ought to do it. Such an approach to ethics largely ignores the personality of the individual that guarantees the actions. Transposing the utilitarianism to physicians’ strike, it is undoubtedly true that strike by physicians result in unbearable suffering of not only the patients in hospitals, but also of the public in general and the nation at large. During physicians’ strike, unnecessary and premature deaths-deaths that could have been prevented are inevitable. According to IRINnews [22], during a 2008 strike by Zimbabwean physicians, Jestina Moyo of Bulawayo, expressed disappointment on arriving at Mpilo central hospital in Bulawayo, with her seriously ill son only to be told that doctors were on strike. She laments:

“*This is painful to watch my son waste away like this. The hospital says the doctors are on strike, demanding high salaries, and there is nothing I can do for my son, as I have no money to take him to a private doctor. As it is, my son will die a painful death unless I find money to take him to a private doctor”*.

According to the same source, since the strike started several death were registered which doctors could have dealt with if they were not on strike. The same consequences have been felt in other countries the world-over. In Malawi, for example, Kelita Kamoto, director of the Queen Elizabeth Central Hospital in Malawi’s largest city Blantyre reported that between 15 and 20 deaths are recorded daily......deaths were registered as the strike entered its third week [23]. In another report by Ecumenical News International (ENI), Nigeria:

“*Constant strikes by Nigerian doctors this year are said to have claimed the lives of more than 20000 patients and have placed a massive burden on Christian hospitals across the country which have been overwhelmed with patients. And other 6000 accident victims died from lack of medical attention as a result of the doctors’ strike”* [24].

In Zambia, The Post Newspaper reported that:

“*Last month, nurses and doctors went on a month-long strike, forcing one Zambian mother to give birth on the sidewalk outside the University Teaching Hospital, the country’s biggest. Her traumatized family took a picture of the ill-fated childbirth, showing the infant’s legs stretching out of the mother, struggling for life -the hospital and potential medical help tantalizingly nearby but completely out of reach. She gave birth without aid from doctors and the newborn died”* [25].

Apart from Zimbabwe, Malawi, Nigeria and Zambia, in the past 20 years there has been strikes by medical doctors in Australia, Belgium, Canada, Chile, Finland, France, Germany, Ghana, India, Ireland, Israel, Italy, Korea, Malta, New Zealand, Peru, Serbia, Spain, Sri Lanka, Romania, USA and UK to name but a few. Many of these strikes have caused lasting damage from which health systems have struggled to get over; have been very costly (both in the short and long term); and have not achieved what the management appear to have wanted.

It can also be argued on the basis of utilitarianism that physicians strike like that of the army, police and prison officers has far reaching consequences to the country in question; may result in violation of human rights and looting of public ‘goods’. One can imagine what may happen if the army, police officers and the prison officers go on strike? If prison officers, for example, go on strike criminals, some with recorded history of mass killing will be free and obviously disturb the harmony of the innocent people. I believe physicians strike causes the same blow to the country involved. It is therefore the contention of this work that just like soldiers, prison officers and police officers who in many countries are not allowed to go on strike, physicians should likewise take no part in any form of strike action.

In view of cases of unnecessary deaths and sufferings of both the patients and the public spelled out in this work, it is undeniable on the basis of utilitarianism that physicians strike has far reaching consequences not only to the patients, but to the public and the national government in question (to the majority). It has been exhibited that the happiness that physician strike brings is clearly overwhelmed by the suffering and sadness it causes to the majority (the patients, public and the government in question). From this understanding the paper contends that physicians strike can never be morally justifiable. Strike fails to achieve a greater happiness to the greatest number of people affected by it.

### Argument from African Communalism

The African scholars surveyed, with the possible exception of Ghanaian philosopher Kwame Gyekye, regard African concepts of the individual and self to be almost totally dependent on and subordinate to social entities and cultural processes [26]. Kenyan theology professor John S. Mbiti, for example, believes that the individual has little latitude for self determination outside the context of the traditional African family and community. He writes:

“Whatever happens to the individual happens to the whole group, and whatever happens to the whole group happens to the individual”. The individual can only say: “I am, because we are; and since we are, therefore I am”; this is a cardinal point in the understanding of the African view of personhood” [27].

Picking it from Mbiti’s understanding, it is clear that the unnecessary suffering of the patients caused by physician strike does not only negatively affect the patients, but also the public and the physicians themselves as members of the group.

Concurring with Mbiti, South African philosophy professor Augustine Shutte, cites the Xhosa proverb: *umuntu ngumuntu ngabantu* (a person is a person through persons) [28]. He elaborates that: This (proverb) is the Xhosa expression of a notion that is common to all African languages and traditional cultures. [29]. It cuts across all African cultures.

For Ghanaian philosopher Kwame Gyekye, the individual, although originating from and inextricably bound to his family and community, nevertheless possesses a clear concept of himself/herself as a distinct person of volition. It is from this combined sense of personhood and communal membership that the family and community expect individuals to take personally enhancing and socially responsible decisions and actions. Although he accepts that the dominant entity of African social order is the community, Gyekye believes:

“*It would be more correct to describe that order as amphibious, for it manifests features of both communality and individuality. African social thought seeks to avoid the excesses of the two exaggerated systems, while allowing for a meaningful, albeit uneasy, interaction between the individual and the society”* [30].

Agreeing with Gyekye, Senegalese philosopher Leopold Senghor regards traditional African society to be based both on the community and on the person and in which, because it was founded on dialogue and reciprocity, the group had priority over the individual without crushing him, but allowing him to blossom as a person [31].

In the same stroke, Achebe commenting on Africa and Africans noted that Africa is not only a geographical expression; it is also a metaphysical landscape-it is in fact a view of the world and of the whole cosmos perceived from a particular position. Achebe goes further to argue that being an African is more than just a matter of passports or of individual volition [32] but of sensibility and responsibility. For Achebe and convincingly so being an African carries penalties. Okot P’Bitek [33] picks up this argument and stresses that in African philosophy, man is not born free to do whatever he wants; in fact it is not even desirable to be so even if it were possible. Thus persons are defined by reference to the environing community, not by focusing on this or that physical or psychological characteristics of the lone individual. This idea has been captured by other several African writers. Mbiti [34], for example, noted that the Africans’ traditional view of person can be summed up in the statement “I am because we are and since we are, therefore I am”. One obvious conclusion to be drawn from this dictum is that personhood in the African traditional context is understood and defined by reference to other members of the same community. A person is defined through other members of his/her community. In this view, physicians in the African context are defined through members of their society (the patients and the public in general). They are physicians because of the people (patients and members of the public) they serve and are part. The relationship between physicians and other members of his/her community (patients and members of the public) can be represented by the syllogism: 1) an individual in the African society is defined through others; 2) A physician (in the African view point) is an individual who lives in a society. Therefore, a physician is defined through other members of his/her society.

As spelled out above, the reality of the communal world takes precedence over the reality of individual life historities, whatever these may be.

In the light of this, it is inescapably true that in the African context and indeed other contexts where people share the same idea of personhood and communal life, physician strike is violation of the public trust-a complete failure to exhibit the prime duty and responsibility to other members of their community. It is thus not only morally unjustifiable but also unfair and unjust to other members of the community. This is so because in any society (where people have the common goals) each member has his duties and responsibilities which s/he should accomplish with all the cogency, dedication and efficiency for his good and the good of the society. This connotes that an African is born with duties and responsibility to his society and the society in turn bestows rights and privileges on its members [35]. The values of individuals and individual rights, for example, are normally overridden by the values and rights of the community as a whole (to which the individual belongs).

## Conclusion

One of the concerns of philosophers of medicine/medical ethicists has been to reflect on the rights, entitlements and obligation of physicians in relation to patients and members of the public. Scientists such as physicians inclined to dealing with factual judgments have failed to convincingly address the issue of strike using the ‘old version’ of the Hippocratic Oath which is the bedrock of the medical profession the world over. Whilst the argument from African communalism are philosophically plausible as has been demonstrated by this work, it is paramount to reiterate that the question of physician strike is too complex to be epitomized in a word. While there are shades of truth in each of the arguments raised in the prior discussion, they are all debatable. One can still argue that physician strikes are morally justifiable since physicians are people with families who need to be financially supported and cared for. Nevertheless, I remain supremely confident that physicians strike is always morally unjustifiable. Like Plato in *The Republic,* and like Aristotle, Aquinas and Dewey, I believe that physicians should through practice, by example, and by the study of ethics learn what it is to be a good physician qua physician, and to practice and value the virtues requisite for good medicine as I have spelled them out in this paper. If any problem, physicians should negotiate peacefully through their associations, courts of law, patients committees, moralists, academics and other stakeholders without contravening the virtues of medicine. They should convince the government or responsible authority for their salaries and conditions of services by arguments and not strike as the latter is detrimental not only to the government/responsible authority, but to patients and members of the public. It is therefore the contention of this paper that to argue that physicians strike is morally unjustifiable is not to say that physicians have no moral rights and entitlements. They do have as they are also human beings who feel, desire and need to be loved and respected by their fellow comrades. However, if problems arise with the employer these should be resolved amicably without losing the essential values of medicine by harming the society (which they are part). In fact, even if it means the revision of medicine’s past to meet its future, this should be done without sacrificing their (physicians) traditional ethical values and causing harm to the patients and the public. By staging a strike the medical fraternity, thus, loses legitimacy and public confidence. After all, when striking the real people who suffer most are the patients and the public, and not the government or the body responsible for their plights and grievances. Physicians are indeed called to work for the people and strikes shrink their professional latitude and diminish them as professionals; hence the need to go back to tradition.

## Competing interests

The author declares no competing interests.
